# Cell differentiation trajectory predicts patient potential immunotherapy response and prognosis in gastric cancer

**DOI:** 10.18632/aging.202515

**Published:** 2021-02-17

**Authors:** Renshen Xiang, Yuping Rong, Yuhang Ge, Wei Song, Jun Ren, Tao Fu

**Affiliations:** 1 Department of Gastrointestinal Surgery II, Renmin Hospital of Wuhan University, Wuhan 430060, Hubei Province, China; 2 The Central Laboratory of the First Clinical College of Wuhan University, Wuhan 430060, Hubei Province, China

**Keywords:** gastric cancer, differentiation trajectory, intratumoral heterogeneity, molecular typing, immune infiltration

## Abstract

The purpose of this study was to investigate the differentiation trajectory of gastric cancer (GC) cells and its clinical relevance and generate a prognostic risk scoring (RS) signature based on GC differentiation-related genes (GDRGs) to predict overall survival (OS). Integrated single-cell RNA sequencing (scRNA-seq) and bulk RNA-seq data from GC samples were used for analysis. The cell differentiation trajectory analysis identified three subsets with distinct differentiation states, of which subsets I/II were involved in metabolic disorders, subset II were also associated with hypoxia tolerance, and subset III were related to immune-related pathways. GC samples were divided into three GDRG-based molecular subtypes, and it was found that molecular typing based on cell differentiation successfully predicted patient OS, clinicopathological features, immune infiltration status, and immune checkpoint gene expression. An eight-GDRG-based prognostic RS signature was generated, and the OS of the high-risk group was significantly worse than that of the low-risk group. By integrating the GDRG-based RS signature with prognostic clinicopathological characteristics, a clinicopathologic-genomic nomogram was constructed, and this nomogram yielded strong predictive performance and high accuracy. The study highlights the implication of GC cell differentiation for predicting patient clinical outcome and potential immunotherapy response and proposes a promising treatment direction for GC.

## INTRODUCTION

Intratumoral heterogeneity is closely associated with tumorigenesis, progression and recurrence; it is an essential characteristic of tumor biological behavior, and novel molecular phenotypes may be generated through different pathogenesis processes, natural evolution and responses to interventions [1]. Molecular phenotypes can reveal a wide range of individual differences related to clinical features, treatment response, tumor resistance, and prognosis [2]. The discovery of tumor-driving genes and drugs targeting these genes provides the possibility of overcoming tumors, but the existence of heterogeneity makes tumor therapy challenging. Therefore, an accurate understanding of tumor heterogeneity to allow the formulation of rational treatment programs has become the focus of precision medicine.

Gastric cancer (GC) is one of the most common gastrointestinal malignancies worldwide and has a high incidence and mortality. According to the latest data from the International Cancer Research Agency, there are approximately 930,000 new cases and 700,000 deaths every year, and GC ranks fourth and second among all malignant tumors in morbidity and mortality, respectively [3]. GC is a heterogeneous tumor, and its biological behavior is influenced by a complicated intracellular gene regulatory network. Traditional pathological typing (e.g., Lauren typing and WHO typing) is no longer sufficient to explain GC heterogeneity. Instead, it is necessary to probe deeply, at the molecular level, to make accurate diagnosis, treatment and prognosis judgments.

As a result of genome and epigenome reprogramming and DNA replication errors during cell division and differentiation, individual cells can present different genomes, transcriptomes and epigenomes [4]. Bulk RNA sequencing (RNA-seq) technology provides the transcription profile of a population of cells or the average expression level of tissues but cannot reveal the gene expression patterns of individual cells [5]. The development of single-cell RNA-seq (scRNA-seq) provides the opportunity for a comprehensive characterization of genetic complexity at the cellular level, including differences in single-nucleotide polymorphisms, copy number variations, gene expression levels, genomic structural variations, gene fusions, alternative splicing and DNA methylation [6], which has contributed to our understanding of cellular heterogeneity. The purpose of this study was to explore the GC cell differentiation trajectory by scRNA-seq and to investigate its relationship with clinical outcomes and potential immunotherapy response in combination with bulk RNA-seq data to provide new insights for GC diagnosis and treatment.

## RESULTS

### Quality control and normalization of scRNA-seq data

**Figure 1:**
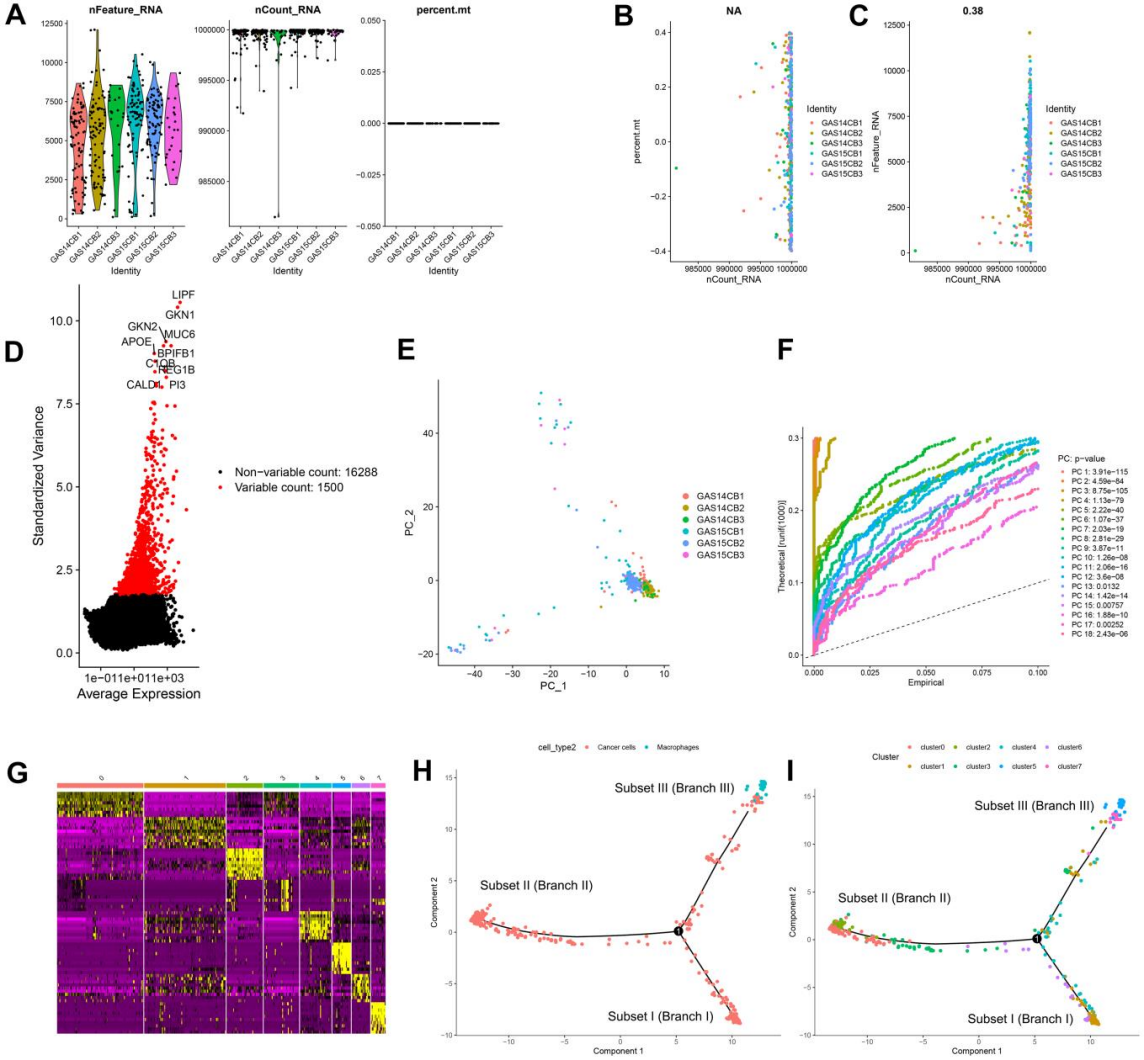
**Quality control and normalization of scRNA-seq data, dimensionality reduction and cell trajectory analysis.** (**A**) After quality control and normalization, 2 nonconforming cells were excluded, and 400 cells were screened for further analysis. (**B**) Correlation analysis between sequencing depth and mitochondrial gene sequences was detected. (**C**) Correlation analysis between sequencing depth and total intracellular sequences. (**D**) A total of 16,288 genes were analyzed, of which 14,788 had low intercellular variation and 1,500 had high variation. (**E**) PCA based on scRNA-seq data. (**F**) Eighteen PCs with significant differences were identified with *P* < 0.5. (**G**) Four hundred GC cells were aggregated into 8 clusters and the top 10% of marker genes in each cluster are displayed on the heat map. (**H**, **I**) Pseudotime and trajectory analysis. PCA: principal component analysis, PCs: principal components, GC: gastric cancer.

In this study, 402 cells from 6 GC samples were obtained from GSE112302. After quality control and normalization, 2 nonconforming cells were excluded, and 400 cells were screened for further analysis (Figure 1A). No correlation between sequencing depth and mitochondrial gene sequences was detected (Figure 1B). There was a significant positive correlation between sequencing depth and total intracellular sequences (R = 0.38, Figure 1C). A total of 16,288 genes were analyzed, of which 14,788 had low intercellular variation and 1,500 had high variation (Figure 1D).

### Cell trajectory analysis identified three GC subsets

Principal component analysis (PCA) was used for preliminary dimensionality reduction of scRNA-seq data. The results showed that there was no significant segregation among GC cells (Figure 1E), so the first 18 principal components (PCs) with significant differences were selected for further analysis (Figure 1F). According to the t-distributed stochastic neighbor embedding (tSNE) algorithm, 400 GC cells were aggregated into 8 clusters, a total of 3339 marker genes were identified by differential analysis, and the top 10% of marker genes in each cluster are displayed on the heat map (Figure 1G). Eight clusters were annotated based on marker genes: clusters 0, 1, 2, 3, 4, 6, and 7 were all cancer cells, and cluster 5 correlated with macrophages. Pseudotime and trajectory analysis indicated that cluster 1 was distributed in subset I, containing cancer cells; clusters 0/2/3 were in subset II, consisting of cancer cells; clusters 5/7 were distributed in subset III, composed of cancer cells and macrophages; and clusters 4/6 were scattered over the three subsets and were also cancer cells (Figure 1H, 1I).

### Molecular functional analysis of three subsets based on GDRGs

**Figure 2:**
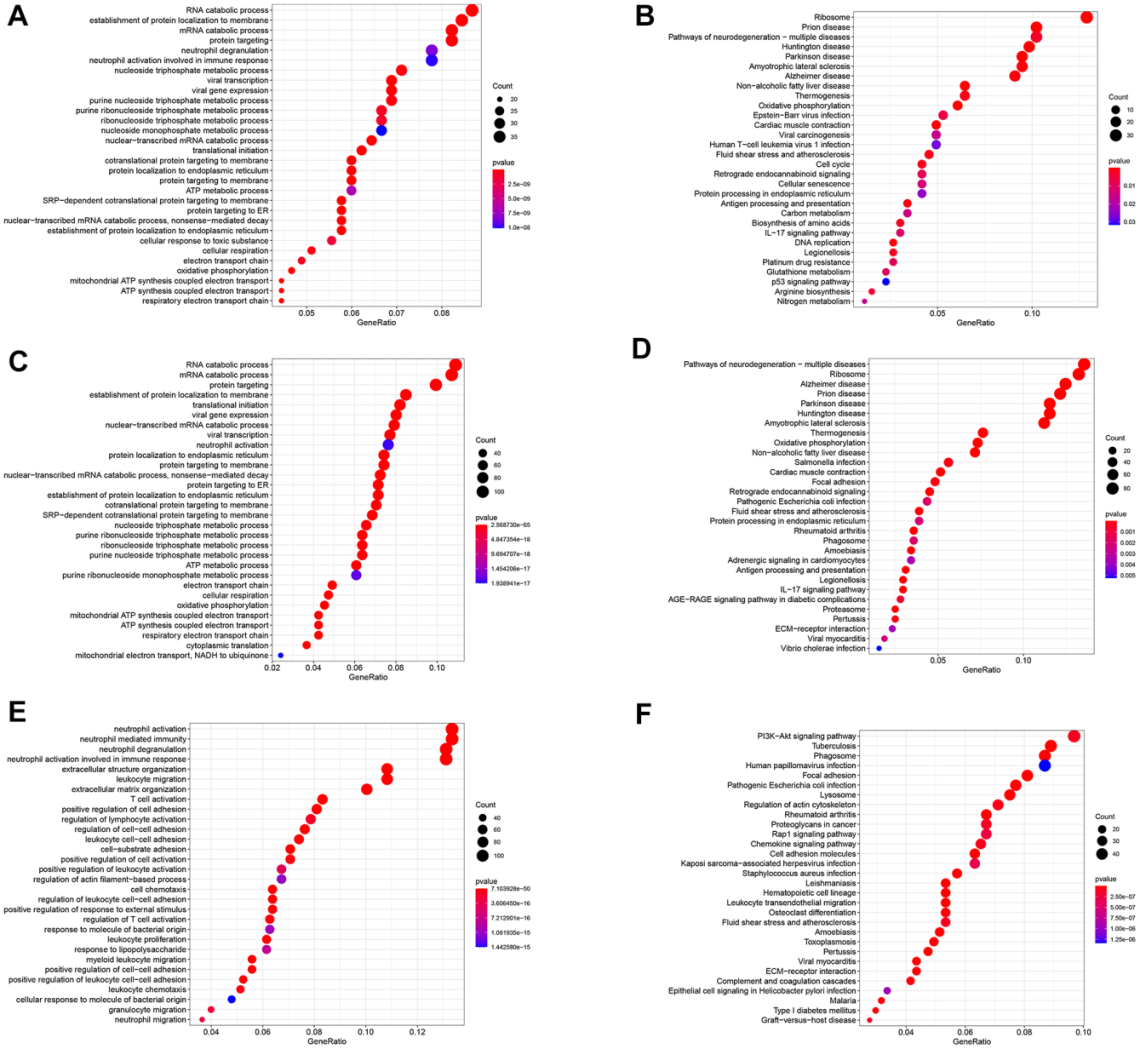
**Functional analysis of three subsets based on GDRGs.** (**A**) GO BP for subset I genes. (**B**) KEGG enrichment analysis for subset I genes. (**C**) GO BP for subset II genes. (**D**) KEGG enrichment analysis for subset II genes. (**E**) GO BP of subset III genes. (**F**) KEGG enrichment analysis of subset III genes. GDRG: gastric cancer differentiation-related gene, GO: Gene Ontology, BP: biological process, KEGG: Kyoto Encyclopedia of Genes and Genomes.

The study identified 402 GC differentiation-related genes (GDRGs) in subset I, 1443 GDRGs in subset II and 1016 GDRGs in subset III. Based on Gene Ontology (GO) biological process (BP) and Kyoto Encyclopedia of Genes and Genomes (KEGG) enrichment analysis, the GDRGs in subsets I/II were involved in mRNA catabolic processes and energy metabolic disorders (Figure 2A–2D), those in subset II were also associated with hypoxia tolerance (Figure 2C, 2D), and those in subset III were closely related to inflammation and immune-related pathways (Figure 2E, 2F).

### Three GDRG-based molecular subtypes of GC patients from the GSE84437 dataset

**Figure 3:**
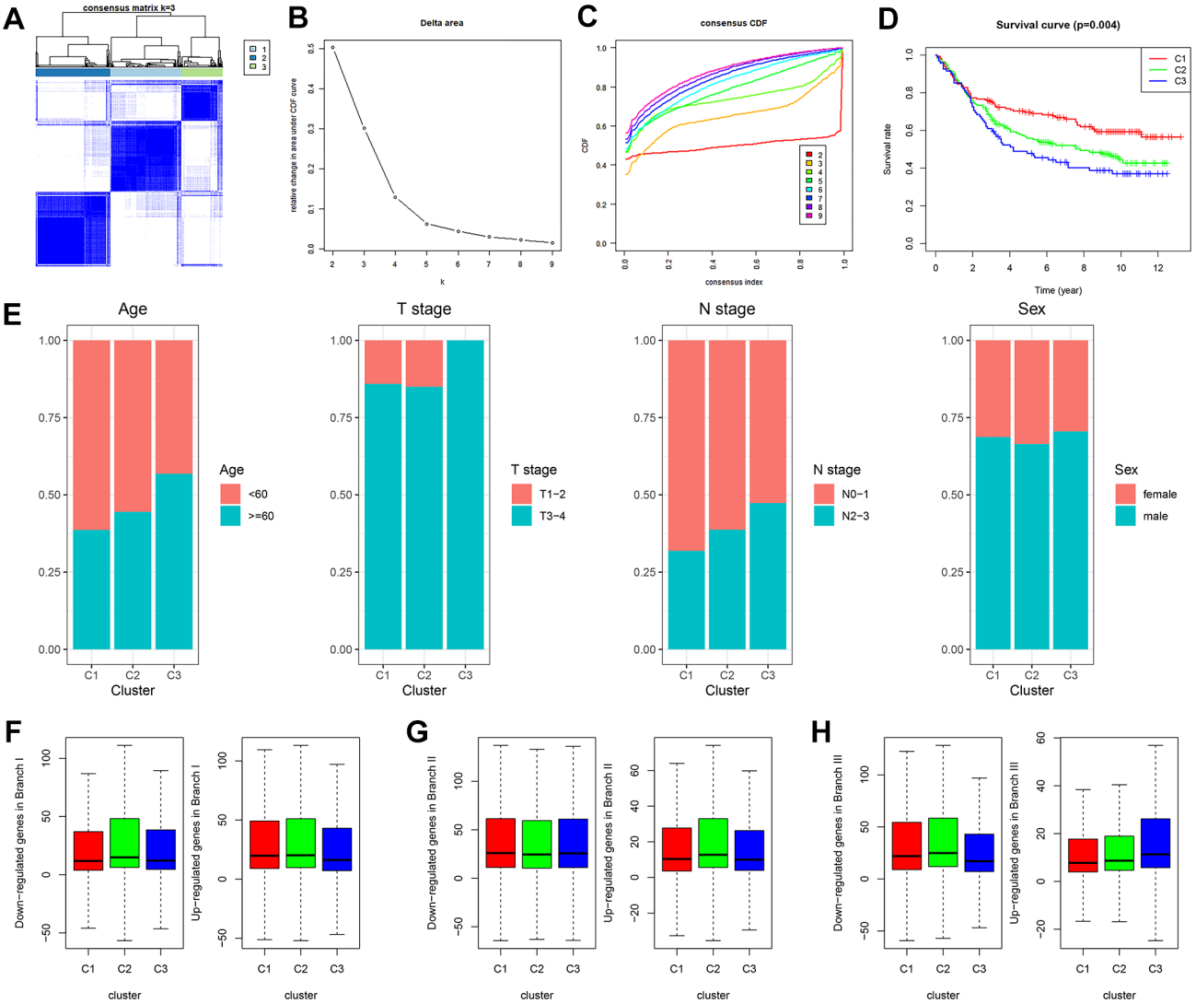
**GDRG-based consensus clustering analysis of GC patients from the GSE84437 dataset.** (**A**–**C**) Three molecular subtypes were identified at a clustering threshold of K = 9. (**D**) Kaplan-Meier analysis between the three molecular subtypes. (**E**) Proportion of clinicopathologic features among the three molecular subtypes. (**F**–**H**) The up/downregulated GDRGs in subsets I/II/III showed the same expression trends as subtypes I/II/III (C1/2/3). GC: gastric cancer, GDRG: GC differentiation-related gene.

GDRG-based consensus clustering analysis was completed in GSE84437, and three molecular subtypes that contained all the GC samples were identified at a clustering threshold of maxK = 9 (Figure 3A–3C). The Kaplan-Meier analysis determined the statistical significance of the consensus clustering results for GC, with subtype I (C1) having the best overall survival (OS), subtype II (C2) having the second best OS, and subtype III (C3) having the worst OS (Figure 3D, *P* = 0.004). From subtype I (C1) to subtype III (C3), patient age decreased, and patients tend to have advanced T stage and N stage, but there was no significant difference in the sex distribution among the three subtypes (Figure 3E and Supplementary Figure 1). In addition, the up/downregulated GDRGs in subsets I/II/III showed the same expression trend in subtypes I/II/III (C 1/2/3) (Figure 3F–3H and Supplementary Figure 1), which indicated that subtypes I/II/III (C 1/2/3) were separately composed of subsets I/II/III.

### Comprehensive analysis of tumor microenvironment scores and immune cell infiltration across three molecular subtypes

**Figure 4:**
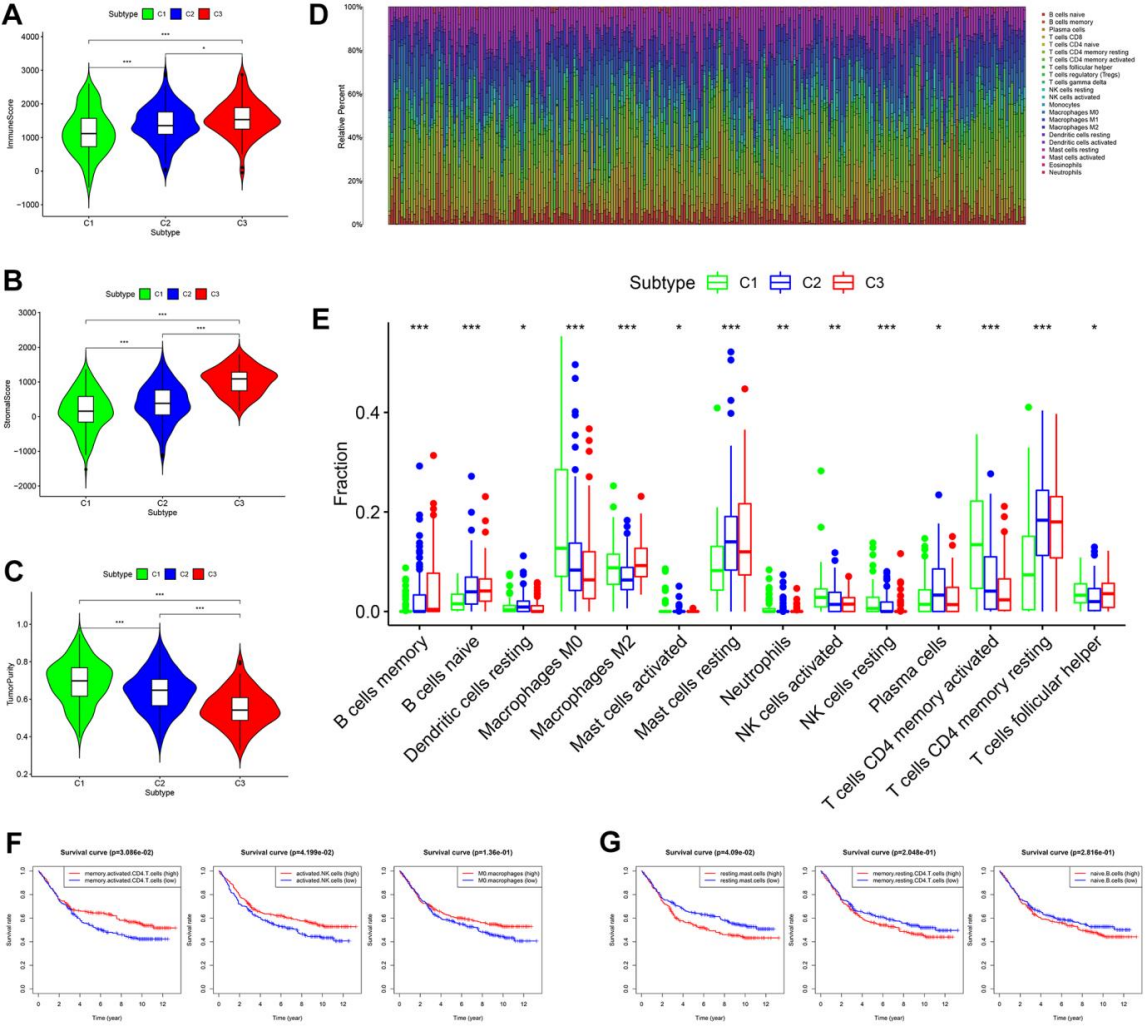
**Comprehensive analysis of tumor microenvironment scores and immune cell infiltration across three molecular subtypes.** (**A**–**C**) Tumor microenvironment scores across three molecular subtypes. (**D**) The contents of 22 immune cells in each sample from the GES84437 dataset. (**E**) Differential analysis showed that the contents of 14 kinds of immune cells were different in the three subtypes. (**F**) Kaplan-Meier analysis of memory-activated CD4^+^ T cells, activated NK cells (*P* < 0.05) and M0 macrophages. (**G**) Kaplan-Meier analysis of resting mast cells, memory resting CD4^+^ T cells and naive B cells.

Based on tumor microenvironment scores, the current study found that immune/stromal scores increased in turn in subtypes I/II/III (C 1/2/3) (Figure 4A, 4B, P < 0.05), while tumor purity gradually decreased in subtypes I/II/III (C 1/2/3) (Figure 4C, P < 0.001). The content of 22 immune cells in each sample was calculated according to the CIBERSORT algorithm and displayed visually such that different colors represent different cell types (Figure 4D). Differential analysis demonstrated that subtype I (C1) contained more memory-activated CD4+ T cells, M0 macrophages, and activated NK cells (Figure 4E, all P < 0.05), which was associated with better OS (Figure 4F). Clusters 2/3 had more memory resting CD4+ T cells, resting mast cells and naive B cells (Figure 4E, all P < 0.05), and the higher infiltration density of these cells was correlated with worse OS (Figure 4G). In addition, the infiltration density of the remaining immune cells (e.g., neutrophils) also showed statistically significant differences in the three subtypes (Figure 4E, all P < 0.05). However, due to the small numbers of these cells in the tumor microenvironment, they were not considered in this study.

### Expression levels of 38 immune checkpoint genes (ICGs) across three molecular subtypes and prognostic analysis

**Figure 5:**
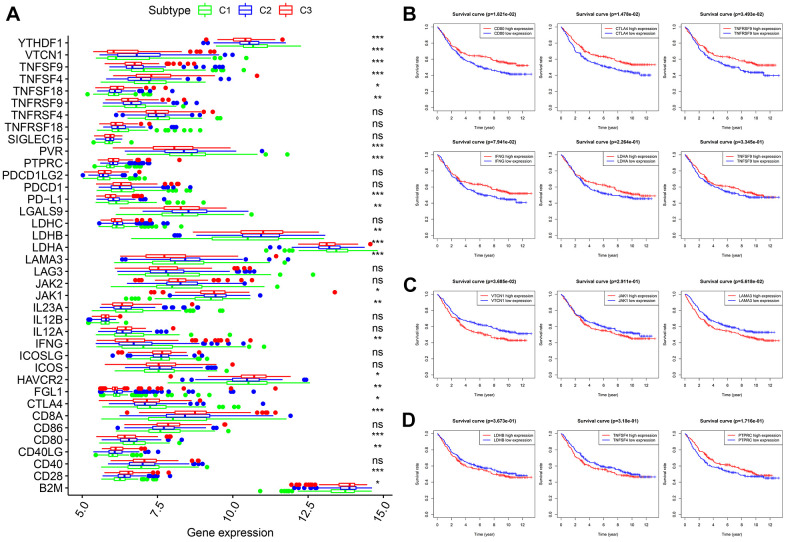
**Expression levels of 38 ICGs across three molecular subtypes and prognostic analysis.** (**A**) Differential expression analysis of 38 ICGs. (**B**) Kaplan-Meier analysis of *CD80*, *CTLA4*, *TNFRSF9*, *IFNG, LDHA* and *TNFSF9*. (**C**) Kaplan-Meier analysis of *VTCN1*, *JAK1* and *LAMA3*. (**D**) Kaplan-Meier analysis of *LDHB*, *TNFSF4* and *PTPRC*. ICGs: immune checkpoint genes.

Thirty-eight confirmed ICGs were obtained from previous studies [7–15]. Through differential expression analysis, we observed significantly high expression of 10 ICGs (namely, CD80, CTLA4, IFNG, IL23A, LDHA, PD-L1, PVR, TNFRSF9, TNFSF9 and YTHDF1) in subtype I (C1) (Figure 5A, all P < 0.05). Importantly, upregulated CD80 (P < 0.05), CTLA4 (P < 0.05), TNFRSF9 (P < 0.05), IFNG, LDHA and TNFSF9 correlated with better OS (Figure 5B). Five ICGs (namely, FGL1, JAK1, LAMA3, LGALS9 and VTCN1) were upregulated in subtype II (C2) (Figure 5A, all P < 0.05), and Kaplan-Meier analysis indicated that highly expressed VTCN1 (P < 0.05), JAK1 and LAMA3 predicted worse OS (Figure 5C). Nine ICGs (namely, B2M, CD28, CD40LG, CD8A, HAVCR2, LDHB, PTPRC, TNFSF18 and TNFSF4) were significantly overexpressed in subtype III (C3) (Figure 5A, all P < 0.05), and Kaplan-Meier analysis showed that LDHB and TNFSF4 expression was associated with poor OS, while PTPRC predicted better OS (Figure 5D). These results not only provide a reasonable molecular basis for the different OS in the three subtypes but also may guide immunotherapy.

### Generation, evaluation and validation of a prognostic risk scoring signature

**Figure 6:**
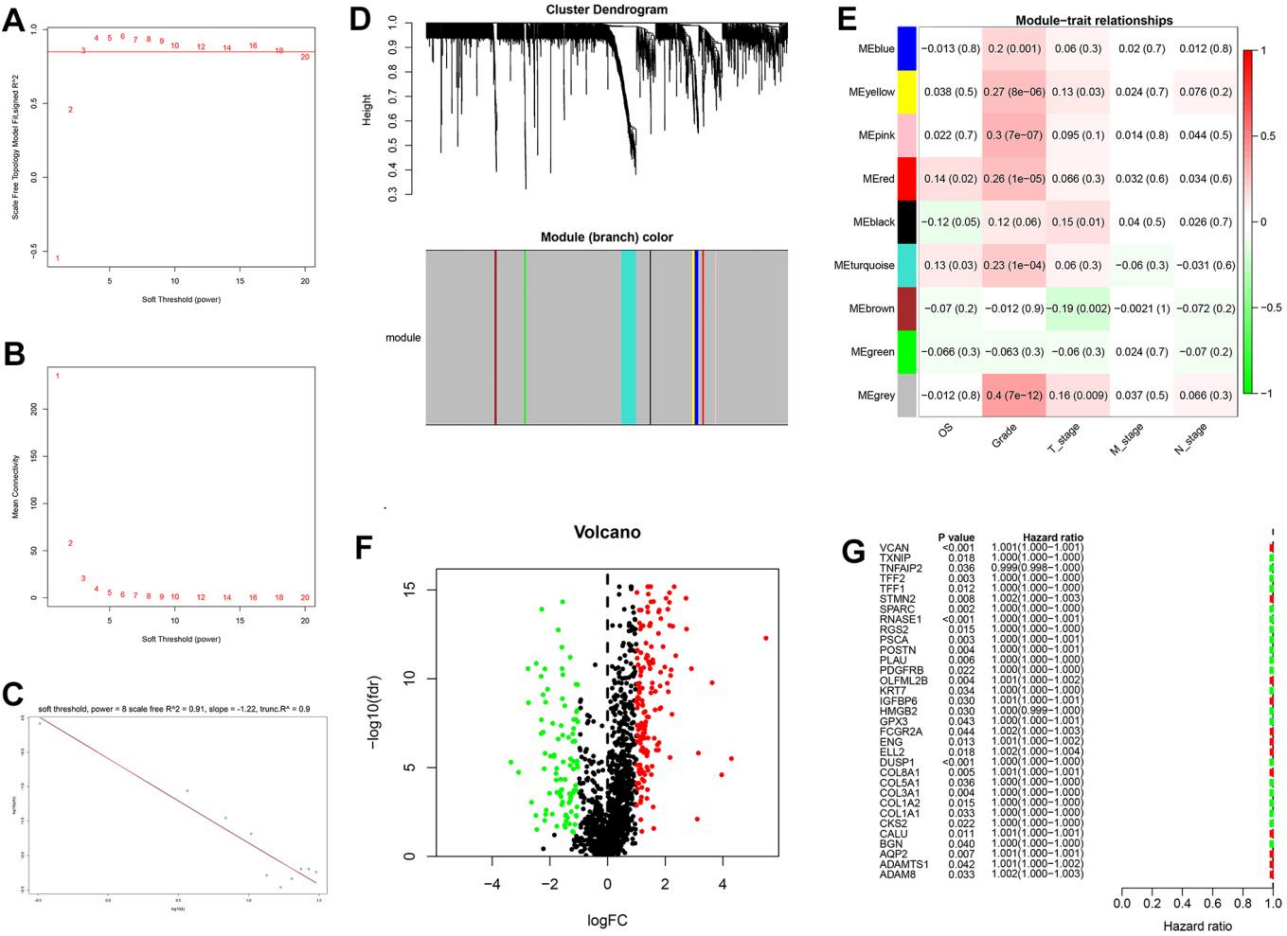
**Weighted correlation network analysis, differential expression analysis and univariate analysis of GDRGs.** (**A**–**D**) Based on weighted correlation network analysis, 9 modules were accessed with a soft threshold = 8. (**E**) Correlation analysis between modules and clinicopathological data. (**F**) Differential expression analysis identified 258 differentially expressed GDRGs in 6 modules, with |log2(FC)| > 1 and FDR < 0.05. (**G**) Univariate analysis of differentially expressed GDRGs. GC: gastric cancer, GDRGs: GC differentiation-related gene, FDR: false discovery rate.

**Figure 7:**
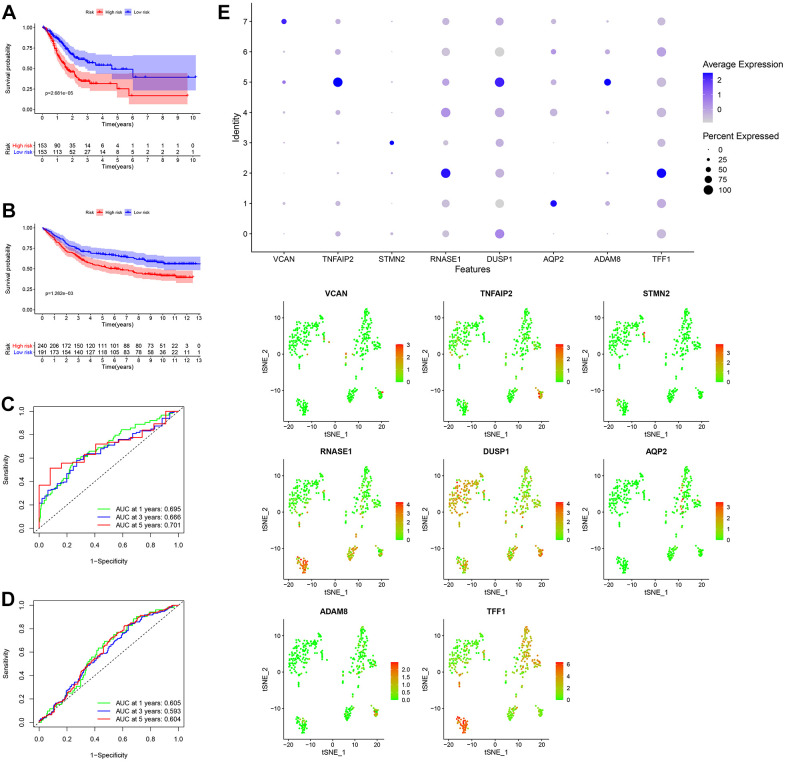
**Generation, evaluation and validation of a prognostic risk scoring signature.** (**A**) Kaplan-Meier analysis between the low-risk group and the high-risk group in the TCGA cohort. (**B**) Kaplan-Meier analysis between the low-risk group and the high-risk group in the GSE84437 dataset. (**C**) In the TCGA cohort, the areas under the ROC curves for predicting 1-year, 3-year and 5-year OS. (**D**) In the GSE84437 dataset, the areas under the ROC curves for predicting 1-year, 3-year and 5-year OS. (**E**) Expression levels of eight GDRGs in eight clusters. OS: overall survival, TCGA: The Cancer Genome Atlas, ROC: receiver operating characteristic, GDRGs: gastric cancer differentiation-related genes.

After the intersection of GDRGs in The Cancer Genome Atlas (TCGA) and GSE84437 cohorts, 1729 GDRGs were enrolled in weighted correlation network analysis (WGCNA). Nine modules were accessed with soft threshold = 8 (Figure 6A–6D), of which 6 modules (namely, blue module, yellow module, pink module, red module, turquoise module and gray module) were closely related to GC grade (Figure 6E). A total of 258 differentially expressed GDRGs (Figure 6F and Supplementary Figure 2) were obtained from 6 modules, and 33 prognostic GDRGs (Figure 6G) were further screened by univariate analysis and incorporated into multivariate Cox regression analysis. Finally, a prognostic risk scoring (RS) signature consisting of 8 GDRGs was generated. The RS of each sample could be calculated according to the relative coefficient and gene expression. RS = (0.00048 * expression of *VCAN*) + (-0.00096 * expression of *TNFAIP2*) + (0.00125 * expression of *STMN2*) + (0.00027 * expression of *RNASE1*) + (0.00013 * expression of *DUSP1*) + (0.00082 * expression of *AQP2*) + (0.00205 * expression of *ADAM8*) + (0.00006 * expression of *TFF1*). According to the prognostic risk scoring signature, the RS of each GC sample in the TCGA and GSE84437 cohorts was calculated, and the OS of the low-risk group was significantly better than that of the high-risk group (Figure 7A, TCGA: *P* = 2.681e-05; Figure 7B, GSE84437: *P* = 1.282e-03). In addition, the areas under the receiver operating characteristic (ROC) curves for predicting 1-year, 3-year and 5-year OS were 0.695, 0.666 and 0.701, respectively, in the TCGA cohort (Figure 7C), and 0.605, 0.593 and 0.604, respectively, in the GSE94437 dataset (Figure 7D).

In addition, the expression levels of eight GDRGs in eight clusters are shown in Figure 7E. *VCAN* increased in cluster 7 (belonging to subset III); *TNFAIP2, DUSP1* and *ADAM8* in cluster 5 (belonging to subset III); *STMN2* in cluster 3 (belonging to subset II); *RNASE1* and *TFF1* in cluster 2 (belonging to subset II); and *AQP2* in cluster 1 (belonging to subset I).

### Establishment and evaluation of a nomogram for predicting patient 3-year and 5-year OS

**Figure 8:**
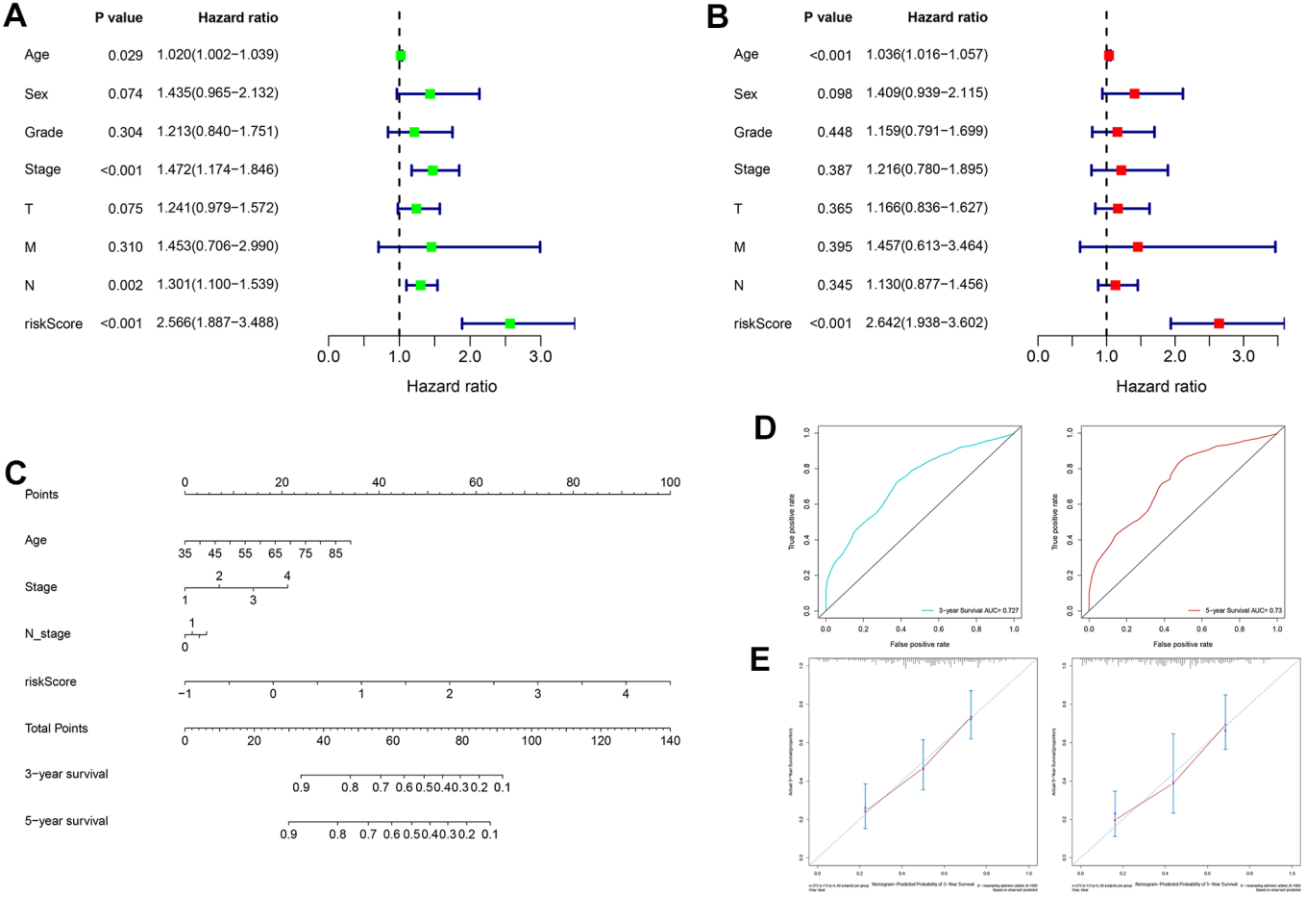
**Construction and evaluation of a nomogram based on the TCGA cohort.** (**A**) Univariate analysis of risk score and clinicopathological characteristics. (**B**) Multivariate analysis of risk score and clinicopathological characteristics. (**C**) A nomogram for predicting 3-year and 5-year OS. (**D**) The areas under the ROC curves for predicting 3-year and 5-year OS. (**E**) The calibration curves for predicting 3-year and 5-year OS. TCGA: The Cancer Genome Atlas, OS: overall survival, ROC: receiver operating characteristic.

In the TCGA cohort, univariate analysis revealed that age, TNM stage, N stage and RS jointly affected patient prognosis (Figure 8A, all *P* < 0.05). Older patients tended to have later clinicopathological stage disease, and higher RS corresponded to poorer prognosis. Subsequently, multivariate analysis demonstrated that age and RS were independent prognostic factors for GC patients (Figure 8B, all *P* < 0.05). Four prognostic factors were combined to construct a nomogram for predicting 3-year and 5-year OS based on the TCGA cohort (Figure 8C). The areas under the ROC curves for predicting 3-year and 5-year OS were 0.727 and 0.730, respectively (Figure 8D). The calibration curves for predicting 3-year and 5-year OS were in good agreement with the observed values (Figure 8E).

## DISCUSSION

In recent years, GC heterogeneity and its implications for the prognosis of patients have been recognized in terms of tumor site (e.g., cardia, gastric body and pylorus) [16], tissue type (e.g., intestinal, diffuse and mixed) [17], pathological type (e.g., papillary adenocarcinoma, mucinous adenocarcinoma, tubular adenocarcinoma and signet ring cell carcinoma), early or advanced stage [18, 19], stage of treatment (e.g., before or after chemotherapy) [20], and primary or metastatic focus [21]. This study further explored GC heterogeneity in terms of the GC cell differentiation trajectory. According to the scRNA-seq data, three subsets with distinct differentiation states were identified, of which the genes characterizing subsets I/II were all involved in mRNA catabolic processes and energy metabolic disorders, those of subset II were also associated with hypoxia tolerance, and those of subset III were closely related to inflammation and immune-related pathways. GDRG-based molecular typing successfully predicted patient OS, clinicopathological features, tumor microenvironment score, immune infiltration status and ICG expression. An eight-GDRG-related prognostic RS signature was generated by multivariate Cox regression analysis, and a nomogram combining RS and clinicopathological characteristics for predicting 3-year and 5-year OS was also constructed.

The development of scRNA-seq technology provides an effective method for revealing the transcriptome characteristics of single cells and opens up a new method for exploring intratumoral heterogeneity [6]. Intratumoral heterogeneity refers to the possibility of acquiring new gene mutations and molecular phenotypes in each progeny after the first cell malignant transformation, so the tumor is a mixture of different cloned tumor cells [2]. In this study, GC cells with distinct differentiation states were projected into three subsets based on cell trajectory analysis, and subset-dependent molecular phenotypes (namely GDRGs) were identified. Molecular functional analysis indicated that the genes characterizing subsets I/II were involved in mRNA catabolic processes and energy metabolic disorders, those of subset II were associated with these processes and with hypoxia tolerance, and those of subset III were closely related to inflammation and immune-related pathways. These results indicated that cell differentiation trajectories can reflect GC heterogeneity and are closely related to metabolic, hypoxia and immune pathways.

Molecular phenotype identification can be used to optimize diagnosis and treatment strategies and promote the development of precision medicine. In 2009, the American Society of Clinical Oncology annual meeting reported that trastuzumab combined with chemotherapy can significantly improve the OS of HER2-positive patients with advanced GC, which was a landmark in GC precision therapy [22]. Apatinib, an anti-VEGFR tyrosinase inhibitor, significantly improves OS in patients with advanced GC [23]. Single-drug ramucirumab, a humanized anti-VEGFR-2 monoclonal antibody, is more effective in the treatment of advanced GC patients than optimal supportive care [24, 25]. Therefore, the GDRGs identified by the GC cell differentiation trajectory are worthy of further study and may be explored as new molecular targets for GC.

With the rapid development of molecular biology and molecular diagnostic technology, GC molecular typing has been improved continuously. For instance, Lei et al [26] classified GC into three independent subtypes (proliferative, metabolic and mesenchymal), which have different molecular and genetic features and different susceptibilities to chemotherapeutic agents. The Cancer Genome Atlas (TCGA) divides GC into four subtypes (namely, EBV-positive, microsatellite instable, genomic stable and chromosomal instable), which provides a roadmap for patient stratifications and trials of targeted therapies [27]. The Asian Cancer Research Group (ACRG) classifies GC subtypes as high microsatellite instable, microsatellite stable/epithelial-mesenchymal transition, microsatellite stable/epithelial/TP53 intact, and microsatellite stable/epithelial/TP53 loss; these subtypes encompass GC heterogeneity and provide useful clinical information [28]. However, the existing GC molecular typing is still immature, and there is no simple correlation between different molecular typing classification schemes, so it is necessary to improve the existing molecular typing or establish new rule sets for specific and accurate molecular typing. In this study, GC was divided into three subtypes based on GDRGs, and the different OS and clinicopathological features among subtypes were reasonably explained by tumor microenvironment scores, immune infiltration status and ICG expression. Abundant lymphocyte infiltration in the tumor microenvironment [29–35] and upregulation of ICGs have been found to be possible reasons for the effectiveness of immunotherapy [36–39], which was consistent with our findings.

GDRG-based GC molecular subtypes showed distinct survival profiles, suggesting that GDRG-based patient classification can be used to predict patient OS. Therefore, a GDRG-based prognostic RS signature was generated, and it was found to have high accuracy and high efficiency. To the best of our knowledge, this is the first GDRG-based signature constructed by multivariate Cox regression analysis. Moreover, a nomogram combining GDRG-based RS and prognostic clinicopathological variables was constructed to provide a visual method for predicting patient OS, which was more accurate and effective than using RS alone.

The current study has several limitations. First, the GDRG-based RS signature was constructed and validated based on retrospective data from TCGA and GEO databases. Further large-scale prospective clinical studies are required to evaluate its effectiveness and practicability. Second, despite the high accuracy and predictive performance of the nomogram, there are additional prognosis-related clinicopathological variables that cannot be accessed from public databases. Therefore, the nomogram includes a limited number of variables and must be further refined at a later stage.

## CONCLUSIONS

The current study identified three subsets of GC cells with distinct differentiation states based on scRNA-seq and demonstrated that GDRG-based molecular typing can accurately predict patient OS, clinicopathological features, tumor microenvironment scores, immune infiltration status and ICG expression. A nomogram combining the CDRG-based RS signature and clinicopathological variables provided an intuitive and accurate method for predicting patient OS. In summary, this study highlights the implications of GC cell differentiation for predicting patient clinical outcome and potential immunotherapy response and proposes a promising treatment direction for GC.

## MATERIALS AND METHODS

### Acquisition and processing of scRNA-seq and bulk RNA-seq

The scRNA-seq data of 402 GC cells in 6 samples were obtained from the GSE112302 dataset in the Gene Expression Omnibus (GEO, https://www.ncbi.nlm.nih.gov/geo/) database. Afterwards, scRNA-seq data were initially processed by the ‘Seurat’ package, the percentage of mitochondrial genes was calculated through the PercentageFeatureSet function, and the relationship between sequencing depth and mitochondrial gene sequences and/or total intracellular sequences was elucidated by correlation analysis. Cells with a gene number < 100, sequencing number < 50 and mitochondrial gene content > 5% were excluded. After data filtering, scRNA-seq data were normalized by the LogNormalize method, and the top 1500 genes with highly variable features were identified by variance analysis. Moreover, the bulk RNA-seq data of 32 normal and 406 GC samples were accessed from TCGA (http://cancergenome.nih.gov/) database, and an additional 433 GC samples were obtained from the GSE84437 dataset (https://www.ncbi.nlm.nih.gov/geo/). The basic clinical information for each sample is detailed in Table 1. In this study, GC samples from patients with a survival time < 30 days, ambiguous survival status or unclear clinicopathological characteristics were excluded.

**Table 1:** Clinicopathological features of patient with gastric cancer.

**TCGA cohort (N = 406)**		**GSE84437 (N = 433)**	
**Variables**	**N (%)**	**Variables**	**N (%)**
**Age (years)**		**Age (years)**	
Mean ± SD*	65.6 ± 10.9	Mean ± SD*	60.1 ±11.6
**Sex**		**Sex**	
Female	150 (36.9)	Female	137 (31.6)
Male	256 (63.1)	Male	296 (68.4)
**Grade**		**Grade**	
I	10 (2.5)	I	-
II	149 (36.7)	II	-
III	240 (59.1)	III	-
unknown	7 (1.7)	unknown	-
**Stage**		**Stage**	
I	56 (13.8)	I	-
II	118 (29.1)	II	-
III	167 (41.1)	III	-
IV	42 (10.3)	IV	-
unknown	26 (6.4)	unknown	-
**T stage**		**T stage**	
T1	23 (5.7)	T1	11 (2.5)
T2	85 (20.9)	T2	38 (8.8)
T3	185 (45.6)	T3	92 (21.2)
T4	103 (25.4)	T4	292 (67.4)
unknown	10 (2.5)	unknown	0 (0.0)
**N stage**		**N stage**	
N0	122 (30.0)	N0	80 (18.5)
N1	109 (26.9)	N1	188 (43.4)
N2	80 (19.7)	N2	132 (30.5)
N3	78 (19.2)	N3	33 (7.6)
unknown	17 (4.2)	unknown	0 (0.0)
**M stage**		**M stage**	
M0	361 (88.9)	M0	-
M1	27 (6.7)	M1	-
unknown	18 (4.4)	unknown	-

*SD: standard deviation.

### Dimensionality reduction and cell annotation

Under the condition of a false discovery rate (FDR) < 0.05, dimensions with significant separation were screened out through PCA [40], and then dimension reduction for the top 18 principal components (PCs) through the tSNE algorithm was employed to obtain the major clusters [41]. With the criteria of log_2_ [fold change (FC)] > 0.5 and FDR < 0.05, marker genes in each cluster were accessed, and the top 10% of marker genes from clusters were laid out in the heatmap. Clusters were annotated through the ‘SingleR’ package based on marker genes.

### Pseudotime and trajectory analysis

Pseudotime and trajectory analyses of GC cells were carried out by the ‘Monocle’ package [42]. Then, intracellular differentially expressed genes in cells with distinct differentiation states with |log2 (FC)| > 0.5 and FDR < 0.05 were designated GDRGs. The ‘clusterProfiler’, ‘org.Hs.eg.db’, ‘enrichplot’ and ‘ggplot2’ packages were used for GO annotation and KEGG enrichment analysis; only BPs were extracted in the GO annotation.

### GDRG-based molecular subtypes of GC patients from the GSE84437 dataset

After log2-scale transformation of GDRG expression in the GSE84437 dataset, 1729 GDRGs were retained for GC molecular typing. The ‘ConsensusClusterPlus’ package, which provided quantitative and visual evidence of stability for measuring the number of unsupervised subtypes, was used for consensus clustering of GC [43]. The K-means algorithm and cumulative distribution function (CDF) curve were utilized to determine the best number of subtypes, and 50 iterations with maxK = 9 were conducted for stable subtypes. Kaplan-Meier analysis was completed to evaluate the survival results, and the ‘ggplot2’ package was used to display the proportion of clinicopathological features in each molecular subtype. Additionally, the expression levels of GDRGs in specific molecular subtypes were investigated within different cell differentiation trajectories and visualized in box plots.

### Tumor microenvironment scores, immune cell infiltration and ICG expression across molecular subtypes

The study calculated the immune/stromal scores and tumor purity of each sample through the ‘ESTIMATE’ package. The content of 22 immune cells in each sample from GSE84437 was identified by CIBERSORT software. Meanwhile, the infiltration density of immune cells in different GC subtypes was compared by the ‘limma’ package, and a histogram of immune cells with significant differences was presented. Furthermore, 38 validated ICGs were summarized through extensive reading of the literature [7–15], and then their expression across GC subtypes was examined by differential expression analysis based on the GSE84437 dataset. Kaplan-Meier analysis was used to investigate the prognostic value of immune cells and ICGs.

### Prognostic risk scoring signature generation and validation

In this study, the TCGA cohort was taken as the training set, and the GSE84437 dataset was taken as the validation set for prognostic RS signature generation and validation. First, the GDRGs in TCGA and GSE84437 cohorts were intersected, and transcription profiles were simultaneously normalized and corrected with log2-scale transformation. Subsequently, GDRGs were included in WGCNA, and key modules related to GC differentiation were identified based on correlation analysis. The genes in key modules were analyzed by differential expression analysis (with |log2(FC)| > 1 and FDR < 0.05) and univariate analysis (*P* < 0.05), and the remaining GDRGs were enrolled in multivariate Cox regression analysis to generate a GDRG-based prognostic RS signature. The mean value of the GDRG-based RS signature can be used to divide patients into high-risk and low-risk groups. The RS can be calculated as the sum of the products of GDRG expression levels and coefficients via the following formula:

$$RS\;=\;\sum_{i}^{k}\left(Exp_{i}\;\times \;Coe_{i}\right)$$

where ‘i’ and ‘k’ represent the ‘i’th gene and total number of genes, respectively. Kaplan-Meier analysis and ROC curves were used to evaluate the accuracy and prediction efficiency.

### Nomogram construction based on TCGA cohort

Based on the TCGA cohort, clinicopathologic variables (e.g., age, sex, tumor grade and TNM staging) and RS were separately incorporated into univariate and multivariate analyses. Subsequently, prognostic variables were combined into a nomogram for predicting 3-year and 5-year OS. ROC curves and calibration curves were used to evaluate the predictive performance and accuracy of the nomogram.

### Statistical analysis

Some continuous variables are expressed as the means ± standard deviation. The χ2 test and T-test/variance analysis were separately used to compare the distribution of dichotomous variables and continuous variables. Kaplan-Meier statistics and log-rank tests were used for survival analysis. All statistical analyses were carried out in R and Perl, and *P* <0.05 was considered to be statistically significant.

### Data accessibility

The scRNA-seq data of GC samples were accessed from GEO database (GSE112302, https://www.ncbi.nlm.nih.gov/geo/). The bulk RNA-seq data of GC samples were obtained from GEO database (GSE84437, https://www.ncbi.nlm.nih.gov/geo/) and TCGA database (http://cancergenome.nih.gov/).

### Ethics approval

Ethical approval was not required because the data came from publicly available databases.

## Supplementary Material

Supplementary Figures
