# Supplementary material for: Cell differentiation trajectory predicts patient potential immunotherapy response and prognosis in gastric cancer
Source: Aging (Albany NY). 2021 Feb 17;13(4):5928–45. doi: 10.18632/aging.202515 (PMC7950306; doi:10.18632/aging.202515)
Supplement: Supplementary Figures [file aging-13-202515-s001.pdf]

SUPPLEMENTARY FIGURES

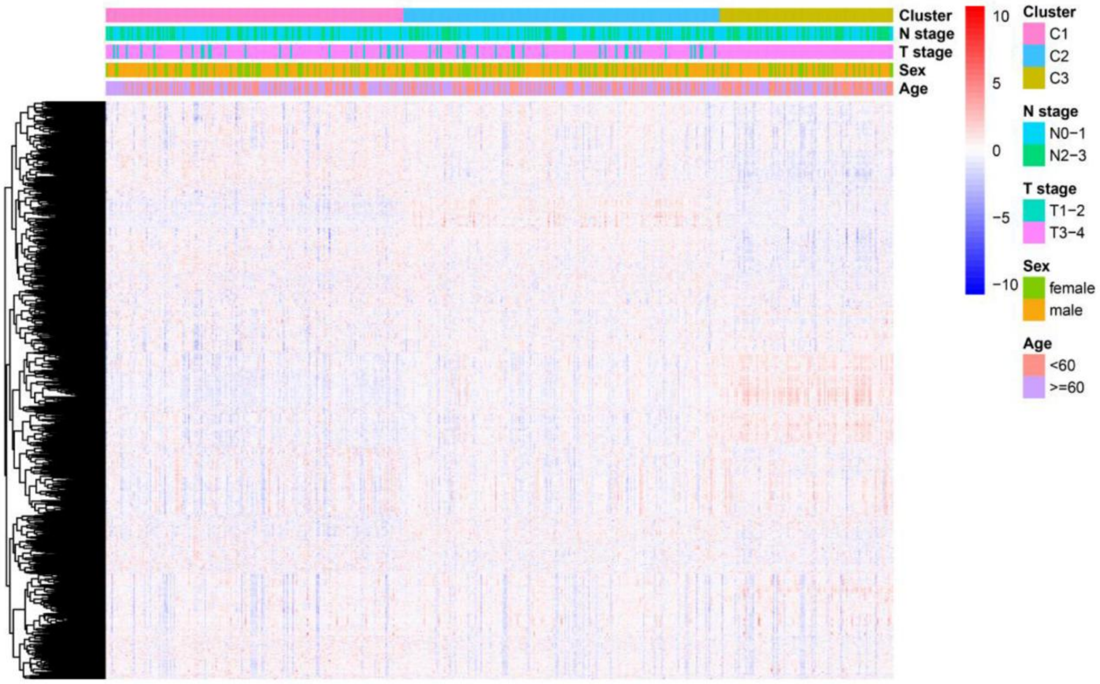

Supplementary Figure 1. Clustering heat map based on the GSE84437 dataset. The expression level of GDRGs and the proportion of clinicopathologic features in three molecular subtypes. GDRG: gastric cancer differentiation-related gene.

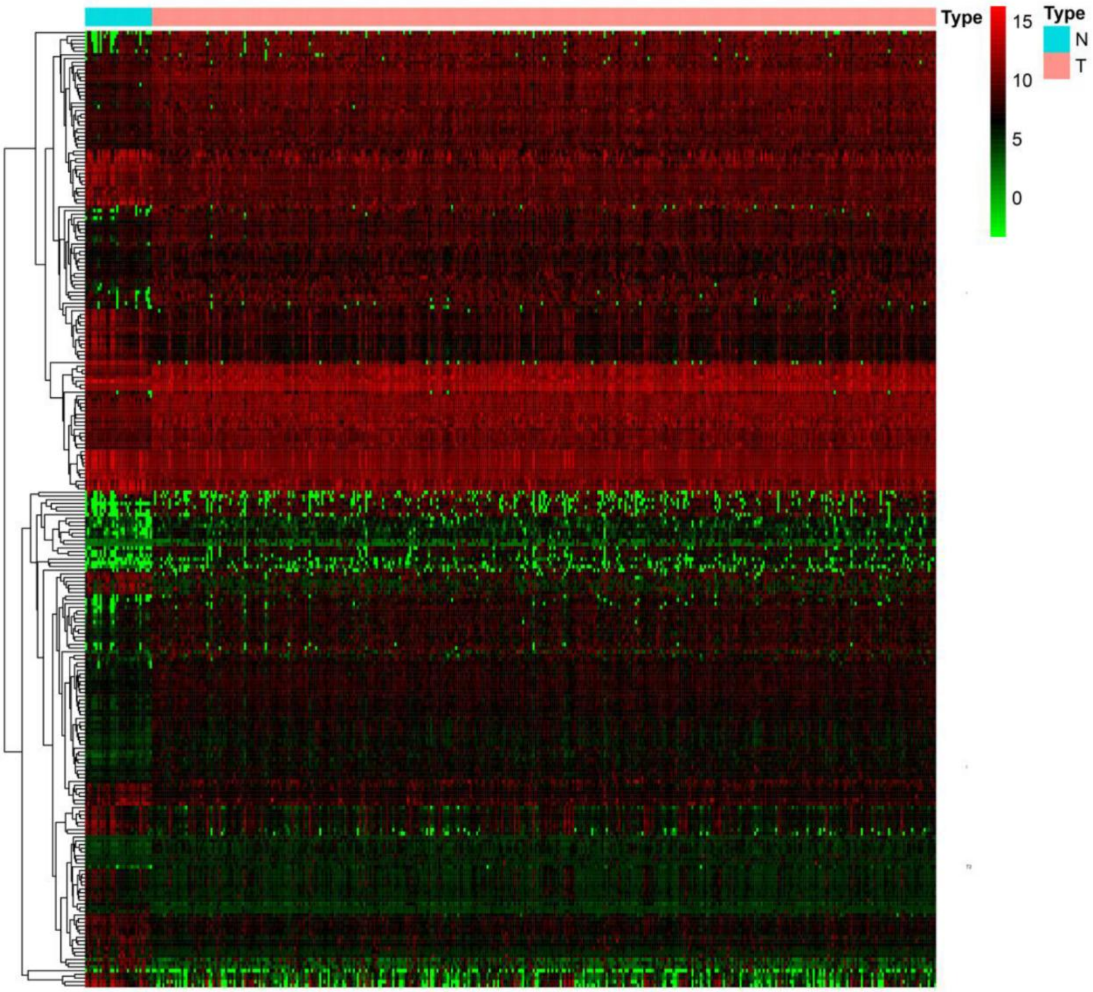

Supplementary Figure 2. Differentially expressed GDRGs based on the TCGA cohort. There were 258 differentially expressed genes between normal and GC samples. TCGA: The Cancer Genome Atlas, GDRG: gastric cancer differentiation-related gene.
